# Molecular Genotyping of *Giardia duodenalis* Isolates from Symptomatic Individuals Attending Two Major Public Hospitals in Madrid, Spain

**DOI:** 10.1371/journal.pone.0143981

**Published:** 2015-12-07

**Authors:** Aida de Lucio, Rocío Martínez-Ruiz, Francisco J. Merino, Begoña Bailo, María Aguilera, Isabel Fuentes, David Carmena

**Affiliations:** 1 Parasitology Service, National Centre for Microbiology, Carlos III Health Institute, Majadahonda, Madrid, Spain; 2 Microbiology and Clinical Parasitology Service, University Hospital Puerta de Hierro Majadahonda, Majadahonda, Madrid, Spain; 3 Microbiology Service, University Hospital Severo Ochoa, Leganés, Madrid, Spain; Aga Khan University Hospital Nairobi, KENYA

## Abstract

**Background:**

The flagellate protozoan *Giardia duodenalis* is an enteric parasite causing human giardiasis, a major gastrointestinal disease of global distribution affecting both developing and industrialised countries. In Spain, sporadic cases of giardiasis have been regularly identified, particularly in pediatric and immigrant populations. However, there is limited information on the genetic variability of circulating *G*. *duodenalis* isolates in the country.

**Methods:**

In this longitudinal molecular epidemiological study we report the diversity and frequency of the *G*. *duodenalis* assemblages and sub-assemblages identified in 199 stool samples collected from 184 individual with symptoms compatible with giardiasis presenting to two major public hospitals in Madrid for the period December 2013–January 2015. *G*. *duodenalis* cysts were initially detected by conventional microscopy and/or immunochomatography on stool samples. Confirmation of the infection was performed by direct immunofluorescence and real-time PCR methods. *G*. *duodenalis* assemblages and sub-assemblages were determined by multi-locus genotyping of the glutamate dehydrogenase (*GDH*) and β-giardin (*BG*) genes of the parasite. Sociodemographic and clinical features of patients infected with *G*. *duodenalis* were also analysed.

**Principal findings:**

Of 188 confirmed positive samples from 178 giardiasis cases a total of 124 *G*. *duodenalis* isolates were successfully typed at the *GDH* and/or the *BG* loci, revealing the presence of sub-assemblages BIV (62.1%), AII (15.3%), BIII (4.0%), AI (0.8%), and AIII (0.8%). Additionally, 6.5% of the isolates were only characterised at the assemblage level, being all of them assigned to assemblage B. Discordant genotype results AII/AIII or BIII/BIV were also observed in 10.5% of DNA isolates. A large number of multi-locus genotypes were identified in *G*. *duodenalis* assemblage B, but not assemblage A, isolates at both the *GDH* and *BG* loci, confirming the high degree of genetic variability observed in other molecular surveys. BIV was the most prevalent genetic variant of *G*. *duodenalis* found in individuals with symptomatic giardiasis in the population under study.

**Conclusions:**

Human giardiasis is an ongoing public health problem in Spain affecting primarily young children under four years of age but also individuals of all age groups. Our typing and sub-typing results demonstrate that assemblage B is the most prevalent *G*. *duodenalis* assemblage circulating in patients with clinical giardiasis in Central Spain. Our analyses also revealed a large genetic variability in assemblage B (but not assemblage A) isolates of the parasite, corroborating the information obtained in similar studies in other geographical regions. We believe that molecular data presented here provide epidemiological evidence at the population level in support of the existence of genetic exchange within assemblages of *G*. *duodenalis*.

## Introduction

*Giardia duodenalis* (syn. *G*. *intestinalis* and *G*. *lamblia*) is a flagellated protozoan parasite belonging to the phylum Sarcomastigophora that colonizes the digestive tract of humans, leading to clinical disease in a significant number of cases. Human giardiasis is a major cause of gastrointestinal illness worldwide, with infections most commonly occurring among children under five years of age and, to a lesser extent, immunocompromised subjects [1]. The disease has been associated with growth and cognitive retardation in children in poor, disadvantaged settings, contributing to the impairment of the socioeconomic development of endemic regions [2]. *G*. *duodenalis* is also the most frequently found protozoa in developed countries, with reported prevalences typically ranging from 2 to 7% depending on the studied population [3]. Giardiasis is mainly acquired via the faecal-oral route following direct contact with infected individuals (e.g. children in day care centres), although waterborne [4] and foodborne [5] outbreaks of human giardiasis are often documented. In addition, production and, to a lesser extent, companion animal species may act directly or indirectly as natural reservoirs of a number of human infections under certain conditions [6,7].

Based on recent molecular and phylogenetic evidence, *G*. *duodenalis* is currently regarded as a species complex consisting of eight (A to H) distinct genetic groups or assemblages with marked differences in host range and specificity [7]. Improvement of genotyping tools has also allowed the detection of further genetic variation within assemblages, resulting in the identification of a number of sub-assemblages [8]. *G*. *duodenalis* assemblages A (~37%) and B (~58%) are responsible of the vast majority of human infections, remaining this proportion approximately constant regardless of the socioeconomic status of the population studied or the geographic area [8]. Other *G*. *duodenalis* assemblages, including C, D, E and F, have been sporadically documented in a number of human isolates, providing molecular evidence of potential zoonotic transmission [7,8].

The global epidemiological situation of *Giardia* in Spain has been recently evaluated [9]. In humans, *G*. *duodenalis* infections have been documented in paediatric [10–12], clinical [13,14], immigrant [15–18], and inmate [19] populations, and also in returning travellers from endemic areas [20,21]. Typical infection rates of giardiasis ranged from 3–7% and 13–25% for asymptomatic and symptomatic individuals, respectively [9]. However, epidemiological information regarding the diversity of *G*. *duodenalis* assemblages and sub-assemblages circulating in Spanish human populations are far scarcer, with few more than 300 isolates typed to date [9].

In this comprehensive molecular epidemiological study we describe the diversity, frequency, and molecular variability at the nucleotide level of *G*. *duodenalis* isolates obtained from symptomatic outpatients and inpatients in two major hospitals in the autonomous region of Madrid, Spain, over a 14-month period of time.

## Methods

### Ethical statement

Patient informed consent was not required as the stool samples used in this study were exclusively intended for routine clinical diagnostic procedures. Gathered or generated socio-demographic or clinical data were conveniently anonymized prior to any analysis to preserve the identity of the patients involved. This study and the procedures involved, including the waiving of informed consent documentation, have been approved by the Research Ethics Committee of the Carlos III Health Institute (reference number: CEI PI 34_2014).

### Population and study design

This is a longitudinal epidemiological survey assessing the presence of *G*. *duodenalis* in patients attending the University Hospitals Puerta de Hierro Majadahonda (UHPHM) and Severo Ochoa (UHSO), two major public hospitals in Madrid, serving an estimated catchment population of 450,000 and 407,000 people, respectively. The study was conducted within a 14 month period from December 2013 to January 2015. Outpatient admissions and inpatients with clinical presentation compatible with giardiasis (acute or persistent diarrhoea, abdominal pain, weight loss, cramps, fever, nausea, and vomiting) were requested to provide a stool sample for parasitological analyses. A limited number of hospitalized cases with any of the symptoms mentioned above were also included. In addition, basic socio-demographic (gender, age, place of birth, history of travelling abroad) and clinical (symptoms, concomitant infections, general immune status, prescribed treatment) data were retrieved from the hospital medical records of each individual case with a presumptive diagnosis of giardiasis.

### Stool samples collection and initial diagnosis of *G*. *duodenalis*

At least a single fresh stool sample was collected per patient. When possible, sequential stool samples from individual patients were pooled. Obtained samples were labelled with anonymized study codes and stored at 4°C until further analyses. The detection of *G*. *duodenalis* was based on conventional microscopy (CM) and/or an immunochromatographic test (ICT). Stool samples were routinely processed at each hospital setting using the concentration systems Para-Pak^®^ PLUS (Meridian Bioscience, Luckenwalde, Germany) or Parasitrap^®^ (Biosepar GmbH, Germany), respectively. Faecal smears were then produced, stained with 1% Lugol's iodine solution, and microscopically examined at 200x magnification, switching to 400x magnification when structures morphologically compatible with *G*. *duodenalis* cysts were suspected. A commercially available lateral flow ICT for the rapid simultaneous detection of *G*. *duodenalis* and *Cryptosporidium* spp. (Cer Test Biotec S.L., Zaragoza, Spain) was also used according to the manufacturer's instructions. From those stool samples that tested positive or probable for *G*. *duodenalis* by conventional microscopy and/or ICT a new, fresh aliquot was sent to the National Centre for Microbiology, Majadahonda (Madrid) for further diagnostic confirmation and genotyping analyses.

### Direct fluorescent antibody test (DFAT)

A direct fluorescent antibody test was used to confirm presumptive microscopy/ICT positive results. Briefly, stool samples (~1 g) were processed using the concentration system PARASEP Midi^®^ (Grifols Movaco, Barcelona, Spain) according to the manufacturer’s instructions. Five μL of concentrated faecal material were placed on welled slides. Smears were air-dried, methanol fixed, and stained with fluorescein-labelled mouse monoclonal antibodies directed against *Cryptosporidium* oocysts and *Giardia* cysts (Crypto/Giardia Cel, Cellabs, Sydney, Australia). Samples were examined on a Zeiss fluorescence microscopy equipped with a MC63 camera system at 400x magnification. Known positive and negative controls were routinely included in each sample batch.

### DNA extraction and purification

Total DNA was extracted from ~200 mg of a new aliquot of faecal material using the QIAamp^®^ DNA stool mini test kit (Qiagen, Hilden, Germany). The extraction was carried out according to the manufacturer’s instructions and the purified DNA (200 μL) was stored at –20°C until further use in downstream molecular analysis.

### Molecular detection of *Giardia duodenalis*

In order to evaluate and compare the diagnostic performance of DFAT, a real-time PCR method was used for the amplification of *G*. *duodenalis* DNA from stool samples [22]. This assay specifically amplified a 62-bp region of the small subunit ribosomal RNA (*SSU* rRNA) gene of the parasite using the primer pair Gd-80F (5´-GACGGCTCAGGACAACGGTT-3´) and Gd-127R (5´-TTGCCAGCGGTGTCCG-3´), and the probe (6-carboxyfluorescein[FAM]-5´-CCCGCGGCGGTCCCTGCTAG-3´-black hole quencher 1 [BHQ1]). Because of its multi-copy nature and expected improvement of PCR sensitivity, this marker is particularly suited for screening purposes in large molecular surveys. Additionally, and based on the highly conserved sequence of the *SSU* rRNA gene, no amplification performance variations were anticipated among *G*. *duodenalis* isolates belonging to different assemblages and sub-assemblages. Amplification reactions were performed in a volume of 25 μL containing 3 μL of template DNA, 12.5 pmol (each) forward and reverse primer, 10 pmol of probe, and 1X TaqMan^®^ Gene Expression Master Mix (Applied Biosystems, California, USA). Following the manufacturer´s recommendations for TaqMan^®^ probes, we used an amplification protocol consisting on an initial hold step of 2 min at 55°C and 15 min at 95°C followed by 45 cycles of 15 s at 95°C and 1 min at 60°C. Amplification and detection of parasitic DNA were performed on a Corbett Rotor-Gene 6000 real-time PCR cycler (Qiagen Corbett, Hilden, Germany) with fluorescence (510 nm) being measured at the end of the annealing step of each cycle. The ramping of the machine was 10°C/s in every step.

### Molecular characterization of *Giardia duodenalis* isolates

*G*. *duodenalis* isolates that tested positive by real-time PCR were subsequently analysed by multi-locus genotyping (MLG) at the glutamate dehydrogenase (*GDH*) and ß-giardin (*BG*) loci. A two-step semi-nested PCR was carried out to amplify a ~432-bp fragment of the *GDH* gene using the primer pairs GDHeF (5´-TCAACGTYAAYCGYGGYTTCCGT-3´) and GDHiR (5´-GTTRTCCTTGCACATCTCC-3´) in the primary reaction, and GDHiF (5´-CAGTACACCTCYGCTCTCGG-3´) and GDHiR in the secondary PCR [23]. PCR reaction mixtures (25 μL) consisted of 5 μL of template DNA, 12.5 pmol (each) forward and reverse primer, a 200 μM concentration of each dNTP, 1.5 mM MgCl_2_, 2.5 units of Taq DNA polymerase (Bioline GmbH, Luckenwalde, Germany), and 1X Reaction Buffer. The primary and secondary PCR reactions were performed in an Applied Biosystems 2720 thermal cycler with a preliminary cycle of 95°C for 3 min, followed by 35 cycles of 95°C for 30 s, 55°C for 30 s and 72°C for 1 min, with a final extension of 72°C. Amplification products were resolved on 2% D5 agarose gels (Conda, Madrid, Spain) stained with Pronasafe nucleic acid staining solution (Conda, Madrid, Spain). Positive-PCR products were then directly sequenced in both directions using the internal primer set described above.

Similarly, a two-step nested PCR was carried out to amplify a ~511-bp fragment of the *BG* gene of G. *duodenalis* using the primer pairs G7_F (5´-AAGCCCGACGACCTCACCCGCAGTGC-3´) and G759_R (5´- GAGGCCGCCCTGGATCTTCGAGACGAC-3´) in the primary reaction, and G99_F (5´-GAACGAACGAGATCGAGGTCCG-3´) and G609_R (5´-CTCGACGAGCTTCGTGTT-3´) in the secondary PCR [24]. PCR reaction mixtures (25 μL) consisted of 3 μL of template DNA, 10 pmol (each) forward and reverse primer, a 200 μM concentration of each dNTP, 1.5 mM MgCl_2_, 2.5 units of Taq DNA polymerase (Bioline GmbH, Luckenwalde, Germany), and 10X Reaction Buffer. The primary PCR reaction was carried out with the following amplification condition: 1 cycle of 95°C for 7 min, followed by 35 cycles of 95°C for 30 s, 65°C for 30 s, and 72°C for 1 min with a final extension of 72°C for 7 min. The conditions for the secondary PCR were identical to the primary PCR except that the annealing temperature was 55°C. PCR products were resolved and sequenced as described above.

### Data analyses

Generated forward and reverse sequences were visually inspected using the Chromas Lite version 2.1 sequence analysis program (http://chromaslite.software.informer.com/2.1/). True or ambiguous polymorphic sites and double peaks were carefully recorded and annotated. Template re-amplification and/or re-sequencing were conducted on a number of DNA isolates to guarantee the accuracy of the sequencing data produced. Sequence similarity searches with sequences deposited in the NCBI and GiardiaDB (http://giardiadb.org/giardiadb/) databases were conducted using the BLAST tool (http://blast.ncbi.nlm.nih.gov/Blast.cgi). Only good quality readings were used to generate consensus sequences at each specific locus. Multiple alignment analyses with appropriate reference sequences obtained from GenBank were conducted by ClustalW in MEGA version 6.0 to determine *G*. *duodenalis* assemblages and sub-assemblages. Phylogenetic trees were constructed based on the neighbour-joining method using the same software [25].

## Results

A total of 199 stool samples from 184 patients with a presumptive diagnosis of giardiasis were included in this survey. CM- and ICT-positive results were obtained for 90.8% (139/153) and 96.0% (191/199) of the stool samples analysed, respectively. Diagnostic results by both methods were produced for a subset of 152 stool samples (S1 Table), as conventional microscopy was not conducted in a total of 47 samples. Overall, 72.4% of the samples (144/199, corresponding to 132 patients) were collected at the UHPHM, and the remaining 27.6% (55/199, corresponding to 52 patients) at the UHSO. These figures represent 1.6% and 1.3% of the total stool samples processed and analysed at the UHPHM and the UHSO, respectively, during the period of study.

### Analysis of socio-demographic parameters

The socio-demographic parameters of symptomatic patients with giardiasis confirmed by real-time PCR or DFAT (see below) are shown in Table 1. The male/female ratio was 1.28. The infection affected mainly children younger than 13 years, particularly those in the age group 0–4 years of age. Adult individuals in the age group 26–50 constituted also a considerable proportion of the cases. Only 7.3% of the infected people were born in other countries including Argentina (1), Bulgaria (1), Chile (2), Colombia (1), Dominican Republic (1), Ecuador (1), Morocco (1), Peru (1), Romania (2), and the Sahrawi Arab Democratic Republic (2).

**Table 1:** Sociodemographic parameters in symptomatic patients with confirmed giardiasis attended at the University Hospitals Puerta de Hierro Majadahonda (UHPHM) and Severo Ochoa (UHSO), Madrid, December 2013–January 2015.

	UHPHM		UHSO		Total	
	Cases N = 128	%	Cases N = 50	%	Cases N = 178	%
**Gender**						
Male	70	54.7	30	60.0	100	56.2
Female	58	45.3	20	40.0	78	43.8
**Age group (years)**						
0–4	48	37.5	19	38.0	67	37.6
5–12	39	30.4	12	24.0	51	28.6
13–25	11	8.6	3	6.0	14	7.9
26–50	24	18.8	13	26.0	37	20.8
>50	6	4.7	3	6.0	9	5.1
**Country of origin**						
Spain	118	92.2	47	94.0	165	92.7
Other European countries	3	2.3	0	0.0	3	1.7
Africa	3	2.3	0	0.0	3	1.7
America	4	3.2	3	6.0	7	3.9
**Travelling abroad**						
No	125	97.7	50	100	175	98.3
Yes	3	2.3	0.0	0.0	3	1.7

### Analysis of clinical parameters

The main clinical parameters of symptomatic patients with giardiasis confirmed by real-time PCR or DFAT (see below) are summarized in Table 2. The clinical manifestations most frequently reported were diarrhoea (75.8%) and abdominal pain (20.8%). The majority (92.7%) of the infected patients did not show any apparent immune debilitating condition, although a number of subjects affected by immunological disorders (2.2%), diabetes (1.7%), or cancer (1.1%) had concomitant intestinal giardiasis. Co-infections with enteric bacterial, protozoan, or helminth pathogens were also detected in a low (≤5%) proportion of individuals. Regarding their consistency, soft stool samples were produced by half (52.3%) of the patients, followed by formed (33.7%) and watery (14.0%) stool samples.

**Table 2:** Clinical parameters in symptomatic patients with confirmed giardiasis attended at the University Hospitals Puerta de Hierro Majadahonda (UHPHM) and Severo Ochoa (UHSO), Madrid, December 2013–January 2015.

	UHPHM		UHSO		Total	
	Cases N = 128	%	Cases N = 50	%	Cases N = 178	%
**Clinical symptoms**						
Diarrhoea	93	72.7	42	84.0	135	75.8
Abdominal pain	29	22.7	8	16.0	37	20.8
Nausea/vomit	14	10.9	0	0.0	14	7.9
Flatulence	2	1.6	0	0.0	2	1.1
Fever	1	0.8	1	2.0	2	1.1
Weight loss	3	2.3	0	0.0	3	1.7
Other^1^	12	9.4	1	2.0	13	7.3
**Immune debilitating conditions**						
None	117	91.4	48	96.0	165	92.7
Immunological disorders^2^	4	3.1	0	0.0	4	2.2
Cancer	1	0.8	1	2.0	2	1.1
Diabetes	3	2.3	0	0.0	3	1.7
AIDS	0	0.0	1	2.0	1	0.6
Other^3^	3	2.3	0	0.0	3	1.7
**Co-infections**						
None	112	87.5	50	100	162	91.0
Bacteria^4^	7	5.5	0.0	0.0	7	3.9
Protozoa^5^	8	6.3	0.0	0.0	8	4.5
Helminths^6^	1	0.8	0.0	0.0	1	0.6
**Stool consistency**						
Formed	56	43.7	4	8.0	60	33.7
Soft	60	46.9	33	66.0	93	52.3
Watery	12	9.4	13	26.0	25	14.0

^1^Patients with other clinical symptoms including anal itching, asthma, bruxism, eosinophilia, and urticaria.

^2^Immunological disorders comprised agammaglobulinemia, allergic reactions, and systemic lupus erythematosus.

^3^Other immune debilitating conditions included asthma, hemochromatosis, hepatic transplant, pregnancy, and spondylosis.

^4^Bacterial pathogens infecting the enteric (*Campylobacter jejuni*, *Clostridium difficile*, *Helicobacter pylori*, *Salmonella* spp., *Yersinia enterocolitica*) or urinary (*Escherichia coli*) tracts of affected patients.

^5^Protozoan species infecting (*Blastocystis hominis*, *Cryptosporidium* spp.) or colonizing (*Endolimax nana*, *Entamoeba coli*) the enteric tract of affected patients.

^6^Infections caused by the enteric helminth parasite *Enterobius vermicularis*.

### Performance of DFAT and real-time PCR for the detection of *G*. *duodenalis*

Overall, out of the 199 stool samples submitted to the National Centre for Microbiology for confirmation purposes, *G*. *duodenalis*-positive results were obtained in 77.5% (148/191) and 94.5% (188/199) of the samples analysed by DFAT and real-time PCR, respectively. Giardiasis was confirmed in 96.7% (178/184) of patients by at least one of these methods. Real-time PCR-positive samples had cycle threshold (Ct) values ranging from 19.5 to 39.6 (mean: 26.3; SD: 3.5). The direct comparison of the diagnostic performance achieved by these methods is shown in S2 Table. A good result correlation was observed in 78.9% (157/199) of the tested samples. A total of 33 cases were diagnosed by real-time PCR but not by DFAT, whereas the opposite was only true for a single sample. These results clearly indicate that real-time PCR was more sensitive than DFAT for the detection of *G*. *duodenalis* in human stool samples. Interestingly, all 10 samples that tested negative by DFAT/real-time PCR originally gave a positive result in ICT, but were negative (*n* = 8) or not examined (*n* = 2) by CM. This finding seems to indicate that, taking DFAT and/or real-time PCR has reference methods, ICT results were associated with a false-positive rate of 5.2% (10/191).

### PCR amplification and initial sequence analysis of *G*. *duodenalis* isolates at the *GDH* and *BG* loci

Successful amplification of the partial *GDH* and *BG* genes was achieved in 60.6% (n = 114) and 47.3% (n = 89), respectively, of the 188 real-time PCR products that tested positive for *G*. *duodenalis*. No amplicons were obtained at these loci with the only sample that tested positive by DFAT but negative by real-time PCR. MLG data were produced for 79 typing results, whereas a total of 35 and 10 isolates were only genotyped at the individual *GDH* or *BG* genes, respectively (S3 Table). Failure to amplify real-time PCR-positive samples at these markers was dependent on the magnitude of Ct values, as demonstrated by the fact that out of the 55 samples with Ct values >28 only 11 (20.0%) and five (9.1%) were successfully typed by *GDH*-PCR and *BG*-PCR, respectively.

To determine the frequency of genotypes at the sub-assemblage level in the studied population, sequence alignment analyses of the obtained *GDH*- and *BG*-PCR products were conducted with appropriate reference sequences (see below). Out of the 114 isolates of *G*. *duodenalis* amplified at the *GDH* locus, one (0.9%) was assigned to the sub-assemblage AI, 30 (26.3%) to AII, five (4.4%) to BIII, 76 (66.7%) to BIV, and two (1.7%) corresponded to discordant genotype results BIII/BIV (S3 Table). Similarly, out of the 89 isolates of *G*. *duodenalis* amplified at the *BG* locus 13 (14.6%) were characterised as sub-assemblage AII (confirming the typing results obtained also at the *GDH* locus), 12 (13.5%) as AIII (11 of them previously assigned to sub-assemblage AII at the *GDH* locus), and 64 (71.9%) as assemblage B, of which 55 were also confirmed at the *GDH* locus (S3 Table). No mixed assemblage A and B infections were detected.

Overall, BIV and AII were the *G*. *duodenalis* sub-assemblages more frequently found in the human population under study, being identified in 62.1% and 15.3% of the isolates analysed (Table 3). Other less prevalent variants included sub-assemblages BIII (4.0%), AI (0.8%), and AIII (0.8%). Typing at the assemblage (B) level was achieved in 6.5% of the isolates. Discordant genotype results AII/AIII or BIII/BIV were identified in 10.5% (13/124) of the isolates characterised (Table 3). No obvious differences in the diversity and frequency of *G*. *duodenalis* assemblages and sub-assemblages were observed between the two hospitals studied.

**Table 3:** Diversity and frequency of *G*. *duodenalis* assemblages and sub-assemblages in symptomatic individuals attended at the University Hospitals Puerta de Hierro Majadahonda (UHPHM) and Severo Ochoa (UHSO) in Madrid, December 2013–January 2015.

	UHPHM		UHSO		Both	
Assemblage/sub-assemblage	Number	%	Number	%	Number	%
AI	1	1.2	0	0.0	1	0.8
AII	13	15.5	6	15.0	19	15.3
AIII	1	1.2	0	0.0	1	0.8
AII/AIII	8	9.5	3	7.5	11	8.9
B	3	3.5	5	12.5	8	6.5
BIII	4	4.8	1	2.5	5	4.0
BIV	52	61.9	25	62.5	77	62.1
BIII/BIV	2	2.4	0	0.0	2	1.6
Total	84	100	40	100	124	100

### Genotyping analysis of *G*. *duodenalis* isolates at the *GDH* locus

Alignment analysis of the only isolate typed as *G*. *duodenalis* sub-assemblage AI showed 100% homology with a 366-bp fragment stretching from positions 103 to 468 of reference sequence L40509. This single sub-type of AI was deposited in GenBank under accession number KT310351. Similarly, 28 out of the 30 isolates assigned to AII were identical to reference sequence L40510 between positions 97 to 461. Two single-nucleotide polymorphisms (SNPs) were identified at positions 99 (C/T) and 292 (G/A), respectively, in the remaining two isolates. Representative sequences of these three variants within sub-assemblage AII were submitted to GenBank under accession numbers KT310352 to KT310354.

Alignment analysis of the five isolates characterized as *G*. *duodenalis* sub-assemblage BIII with reference sequence AF069059 allowed the identification of a 390-bp stretch, equivalent to positions 61–450 of AF069059. All five BIII isolates differed among them by one to four SNPS, and by six to ten SNPs when compared with the selected reference sequence. Transition (C ↔ T or A ↔ G) mutations were responsible for 95.1% (39/41) of the SNPs detected, whereas transversion (purine ↔ pyrimidine) mutations were found in the remaining two cases. Double peaks accounted for 82.9% (34/42) of the SNPs identified during the chromatogram reads. Additionally, only 4.9% (2/41) of the point mutations described were directly associated to amino acid changes at the protein level (Table 4). The sequences (one novel and four known) of these isolates were submitted to GenBank under accession numbers KT310355 to KT310359.

**Table 4:** Diversity and frequency of single-nucleotide polymorphisms displayed by *Giardia duodenalis* sub-assemblage BIII isolates at the glutamate dehydrogenase locus (partial sequence between positions 61 to 450) identified in the present study. Novel genotypes were shown underlined. Point mutations inducing amino acid substitutions were highlighted as superscript numbers indicating the amino acid change. Transversion mutations were highlighted in bold.

		Nucleotide at position of reference sequence AF069059																			
		87	99	132	147	150	162	174	219	237	247	270	276	309	330	336	341	351	381	387	402
		C	C	C	T	G	T	C	T	T	A	C	T	C	C	C	G	C	G	C	G
Isolate	Number of isolates																				
KT310355	1	.	.	Y	Y	R	Y	Y	.	C	.	**M** ^1^	.	T	.	Y	.	.	.	.	R
KT310356	1	.	T	.	Y	R	.	.	.	Y	.	.	.	Y	Y	Y	.	.	.	.	R
KT310357	1	.	Y	.	Y	.	.	.	Y	Y	**M**	.	.	Y	Y	.	.	Y	.	Y	R
KT310358	1	Y	.	.	Y	R	.	.	.	.	.	.	.	T	.	Y	R	.	R^2^	.	.
KT310359	1	.	Y	.	Y	R	.	.	.	.	.	.	C	T	.	T	.	.	.	.	.

M: A/C; R: A/G; Y: C/T.

^1^p.D90E.

^2^p.R114K.

Similarly, multiple sequence alignment analyses of the 76 DNA isolates assigned to *G*. *duodenalis* BIV revealed a significant degree of genetic heterogeneity at the nucleotide level, allowing their allocation into five distinct clusters (Table 5). The sequences of nearly half (37/76) of the isolates analysed were identical to the stretch comprising positions 102 to 479 of reference sequence L40508, while the remaining half varied by one to seven SNPs. All 53 SNPs detected corresponded to transition mutations, with double peaks appearing in 60.4% (32/53) of them. Only a single point mutation led to an amino acid change within the protein chain (Table 5). Representative BIV sequences, including a novel genotype, were deposited in GenBank under accession numbers KT310360 to KT310373.

**Table 5:** Diversity and frequency of single-nucleotide polymorphisms displayed by *Giardia duodenalis* sub-assemblage BIV isolates at the glutamate dehydrogenase locus (partial sequence between positions 102 to 479) identified in the present study. Novel genotypes were shown underlined. Point mutations inducing amino acid substitutions were highlighted as superscript numbers indicating the amino acid change.

		Nucleotide at position of reference sequence L40508															
			135	153	183	186	203	273	288	351	352	366	387	396	423	438	462
			T	G	T	G	G	C	C	T	C	T	T	C	C	A	T
Isolate	Number of isolates	Cluster															
KT310360	37	1	.	.	.	.	.	.	.	.	.	.	.	.	.	.	.
KT310361	1	1	.	.	.	.	.	.	Y	.	.	.	.	.	.	.	.
KT310362	1	2	.	.	C	A	.	T	.	.	.	.	C	T	T	G	.
KT310363	17	3	.	.	C	.	.	.	.	.	.	.	C	T	T	.	.
KT310364	1	3	.	R	C	.	.	.	.	.	.	.	C	T	T	.	.
KT310365	1	3	.	.	C	.	.	.	.	.	.	.	Y	Y	T	.	.
KT310366	1	3	.	.	C	.	.	.	.	.	.	.	Y	Y	Y	.	.
KT310367	1	3	.	.	Y	.	.	.	.	.	.	.	C	T	T	.	.
KT310368	1	3	.	.	Y	.	.	.	Y	.	.	.	Y	Y	Y	.	.
KT310369	11	3	.	.	Y	.	.	.	.	.	.	.	Y	Y	Y	.	.
KT310370	1	3	.	.	.	.	.	.	.	.	.	.	Y	Y	Y	.	.
KT310371	1	3	Y	.	Y	.	.	.	.	.	Y	.	Y	Y	Y	.	.
KT310372	1	4	.	.	.	.	R^1^	.	Y	.	.	.	.	.	.	.	Y
KT310373	1	5	.	.	.	.	.	.	.	Y	.	Y	Y	.	.	.	.

R: A/G; Y: C/T.

^1^p.G68D.

Two additional *G*. *duodenalis* isolates produced discordant sequence genotyping results BIII/BIV. Both isolates were independently aligned to reference sequences AF069059 (BIII) or L40508 (BIV), leading to the identification of seven to 15 SNPs mostly associated to the presence of double peaks (S4 and S5 Tables). BIII/BIV sequences were submitted to GenBank under accession numbers KT310374 to KT310375.

**Fig 1:**
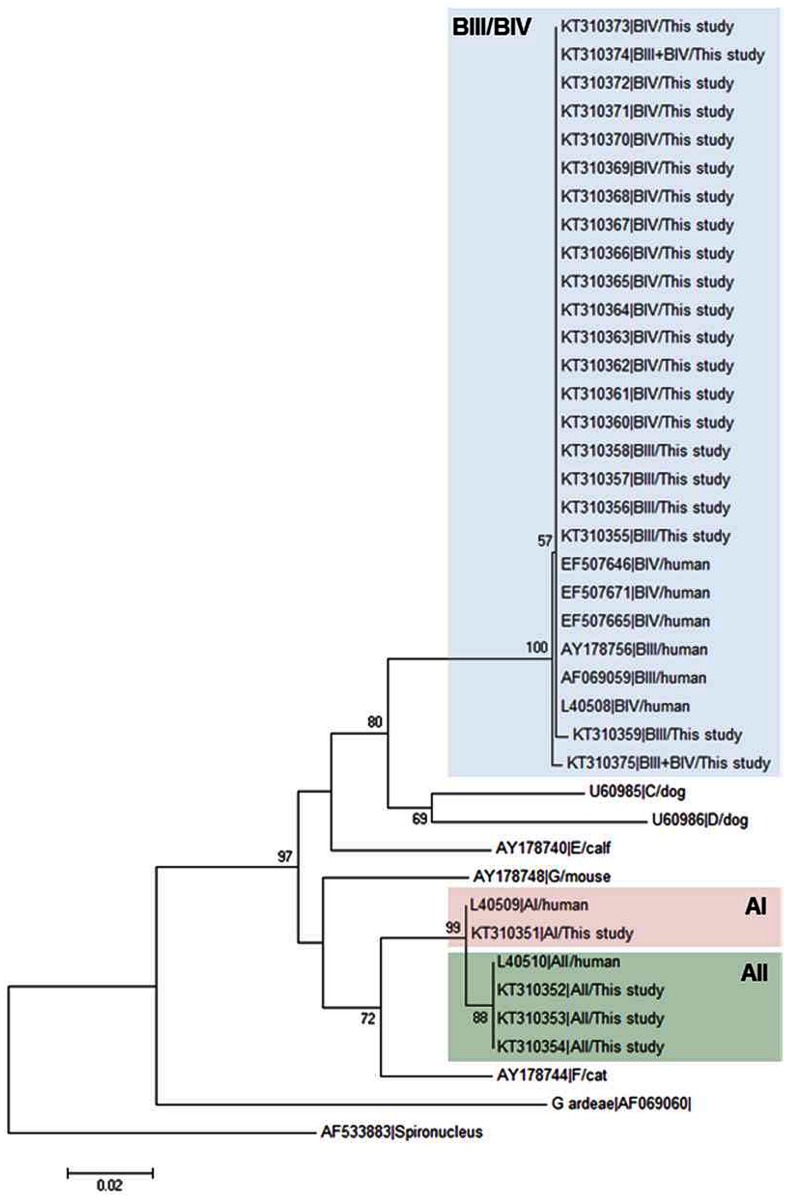
Evolutionary relationships among assemblages of *G*. *duodenalis* at the *GDH* locus inferred by a neighbour-joining analysis of the nucleotide sequence covering a 359-bp region (positions 103 to 461 of GenBank accession number L40508) of the gene. GenBank accession numbers are provided for each sequence used. The bootstrap consensus tree was inferred from 1,000 replicates. Branches corresponding to partitions that were reproduced in less than 50% of bootstrap replicates are collapsed. The bootstrap values are indicated at the branch points. The evolutionary distances were computed using the Kimura 2-parameter method. The rate variation among sites was modelled with a gamma distribution (shape parameter = 2). *Spironucleus vortens* was used as outgroup taxa.

Fig 1 shows the neighbour-joining tree produced from the alignment of the *GDH* partial sequences obtained in this study and representative reference sequences retrieved from GenBank. All BIII and BIV isolates fell within an evolutionary conserved cluster (as demonstrated by the very short length of the branches) but without forming discrete groups. On the contrary, AII and AIII isolates grouped together in independent clusters.

### Genotyping analysis of *G*. *duodenalis* isolates at the *BG* locus

Out of the 13 isolates of *G*. *duodenalis* assigned to the sub-assemblage AII at the *BG* locus, 11 were identical to reference sequence AY072723 between positions 99 to 605, whereas the remaining two isolates constituted distinct (one novel and one known) genotypes showing SNPs at positions 423 (T/C) and 436 (A/R), respectively. Representative sequences of these genetic variants of sub-assemblage AII were deposited in GenBank under accession numbers KT310376 to KT310378. Similarly, very little genetic variability was observed among the 12 isolates typed as AIII. Alignment analyses allowed the identification of a 490-bp fragment corresponding to positions 116 to 605 of reference sequence AY072724.1. A total of 10 isolates showed 100% sequence identity with AY072724.1 at the compared stretch, with an additional two isolates (one novel and one known) differing by a single SNP at positions 375 (C/Y) and 537 (G/A), respectively. Representative sequences of these three AIII genotypes were submitted to GenBank under accession numbers KT310379 to KT310381.

Paralleling the results obtained at the *GDH* locus, multiple sequence alignment analysis of the 64 isolates genotyped as assemblage B at the *BG* locus revealed a high level of genetic polymorphism, as illustrated by the fact that none of them had 100% identity with reference sequence AY072727.1 between positions 107 to 575. A total of 25 (2 novel and 23 known) genotypes, varying from one to 13 SNPs among them, were identified. Based on the nucleotide diversity patterns observed, these DNA isolates were grouped into seven clusters (Table 6). Transition and transversion mutations were associated to 98.6 (n = 145) and 1.4% (n = 2) of the 147 SNPs detected, respectively. Double peaks were identified in 28.6% (n = 42) of these SNPs, with 9.5% (n = 14) of them being linked to amino acid changes in the protein chain (Table 6). Representative assemblage B sequences, including two novel genotypes, were deposited in GenBank under accession numbers KT310382 to KT310406.

**Table 6:** Diversity and frequency of single-nucleotide polymorphisms displayed by *Giardia duodenalis* assemblage B isolates at the beta giardin locus (partial sequence between positions 107 to 575) identified in the present study. Novel sub-types were shown underlined. Point mutations inducing amino acid substitutions were highlighted as superscript numbers indicating the amino acid change. Transversion mutations were highlighted in bold.

			Nucleotide at position of reference sequence AY072727.1																																				
			126	132	133	159	165	171	183	186	189	218	228	240	256	258	274	283	309	324	325	340	345	347	354	393	426	427	453	454	455	456	471	484	485	519	520	543	564
			C	T	G	G	C	A	A	G	G	T	A	T	C	G	A	A	C	C	A	G	G	A	G	C	C	G	C	G	A	G	T	G	A	T	G	C	C
Isolate	Number of isolates	Cluster																																					
KT310382	1	1	T	.	.	.	.	.	.	.	.	.	.	.	.	.	.	.	.	.	.	.	.	.	.	.	.	.	.	.	.	.	.	.	.	.	.	.	.
KT310383	1	1	.	.	.	.	.	.	.	.	.	.	.	.	.	.	.	.	T	.	.	.	.	.	.	.	.	.	.	.	.	.	.	.	.	.	.	.	.
KT310384	1	1	.	.	.	.	.	.	.	.	.	.	.	.	.	.	.	.	Y	.	.	.	.	.	.	.	.	.	.	.	.	.	.	.	.	Y	.	.	.
KT310385	1	2	.	.	A^1^	A	T	.	.	.	.	.	.	.	.	.	.	.	T	T	.	.	.	.	.	T	.	.	.	.	.	.	C	.	.	.	.	.	.
KT310386	35	2	.	.	.	A	T	.	.	.	.	.	.	.	.	.	.	.	T	T	.	.	.	.	.	T	.	.	.	.	.	.	C	.	.	.	.	.	.
KT310387	1	2	.	.	.	A	T	.	.	R	.	.	.	.	.	.	.	.	T	T	.	.	.	.	.	T	.	.	.	.	.	.	C	.	.	.	.	.	.
KT310388	1	2	.	.	.	A	T	.	.	.	.	Y^2^	.	.	.	.	.	.	T	T	.	.	.	.	.	T	.	.	.	.	.	.	C	.	.	.	.	.	.
KT310389	1	2	.	.	.	A	T	.	.	.	.	.	.	.	Y	.	.	.	T	T	.	.	.	.	.	T	.	.	.	.	.	.	C	.	.	.	.	.	.
KT310390	1	2	.	.	.	A	T	.	.	.	.	.	.	.	.	.	R^4^	.	T	T	.	.	.	.	.	T	.	.	.	.	.	.	C	.	.	.	.	.	.
KT310391	1	2	.	.	.	A	T	.	.	.	.	.	.	.	.	.	.	.	T	T	R^6^	.	.	.	.	T	.	.	.	.	.	.	C	.	.	.	.	.	.
KT310392	1	2	.	.	.	A	T	.	.	.	.	.	.	.	.	.	.	.	T	T	.	.	.	R^8^	.	T	.	.	.	.	R^11^	.	C	.	.	.	.	.	.
KT310393	1	2	.	.	.	A	T	.	.	.	.	.	.	.	.	.	.	.	T	T	.	.	.	.	.	T	.	.	.	.	.	.	C	.	.	Y	.	.	.
KT310394	1	2	.	.	.	R	T	.	.	.	.	.	.	.	.	.	.	.	T	T	.	.	.	.	.	T	.	.	.	.	.	.	C	.	.	.	.	.	.
KT310395	2	2	.	.	.	R	T	.	.	.	.	.	.	.	.	.	.	.	T	Y	.	.	.	.	.	T	.	.	.	.	.	.	Y	.	.	.	.	.	.
KT310396	2	2	.	.	.	.	T	.	.	.	.	.	.	.	.	.	.	.	T	T	.	.	.	.	.	T	.	.	.	.	.	.	C	.	.	.	.	.	.
KT310397	1	2	.	.	.	.	T	.	.	.	.	.	.	.	.	.	R^4^	.	T	T	.	.	.	.	.	T	.	.	.	.	.	.	C	.	.	.	.	.	.
KT310398	1	2	.	.	.	.	T	.	.	.	.	.	.	.	.	.	.	.	T	T	.	.	.	.	.	T	Y	.	.	.	.	.	C	.	.	.	.	.	.
KT310399	1	2	.	.	.	.	T	.	.	.	.	.	.	.	.	.	.	.	T	T	.	.	.	.	.	T	.	.	.	.	.	.	C	.	.	.	R^14^	.	.
KT310400	4	2	.	.	.	.	T	.	.	.	.	.	.	.	.	.	.	.	T	.	.	.	.	.	.	T	.	.	.	.	.	.	.	.	.	.	.	.	.
KT310401	1	2	.	.	.	.	T	.	.	.	.	.	.	.	.	.	.	.	T	.	.	.	.	.	.	T	.	.	.	.	.	.	.	.	R^13^	.	.	.	.
KT310402	1	3	.	Y	.	A	Y	R	R	.	.	.	R	.	.	R	.	.	T	T	.	.	.	.	.	T	.	.	**M**	.	.	R	C	.	.	.	.	.	.
KT310403	1	4	.	.	.	.	Y	.	R	.	R	.	.	.	.	.	.	.	T	Y	.	.	.	.	.	Y	.	.	.	.	.	.	.	.	.	Y	.	.	Y
KT310404	1	5	.	.	.	.	T	.	.	.	.	.	.	.	.	.	.	.	T	.	.	R^7^	R	.	.	.	.	R^9^	.	R^10^	.	.	.	R^12^	.	.	.	T	.
KT310405	1	6	.	.	.	.	.	.	R	.	.	.	.	Y^3^	**M**	.	.	R^5^	Y	.	.	.	.	.	R	.	.	.	.	.	.	.	.	.	.	.	.	.	.
KT310406	1	7	.	.	.	.	.	.	G	.	.	.	.	.	.	.	.	.	T	.	.	.	.	.	.	.	.	.	.	.	.	.	.	.	.	C	.	.	T

M: A/CA; R: A/G; Y: C/T.

^1^p.V47M.

^2^p.I75T.

^3^p.L88M.

^4^p.I94V.

^5^p.M97V.

^6^p.T111A.

^7^p.E116K.

^8^p.N118S.

^9^p.E145K.

^10^p.E154K.

^11^p.E154G.

^12^p.E164K.

^13^p.E164G.

^14^p.A176T.

**Fig 2:**
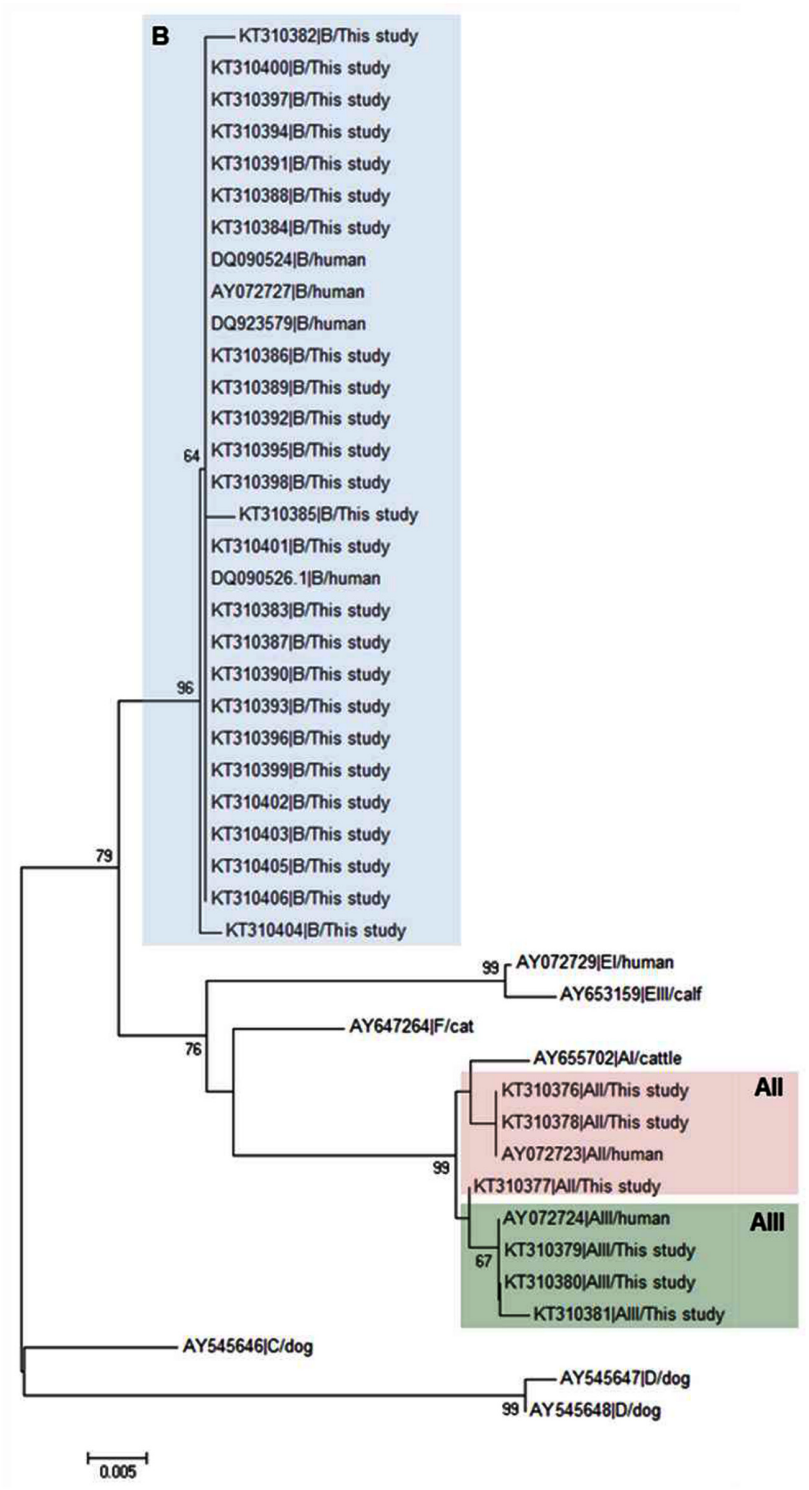
Evolutionary relationships among assemblages of *G*. *duodenalis* at the *BG* locus inferred by a neighbour-joining analysis of the nucleotide sequence covering a 458-bp region (positions 116 to 573 of GenBank accession number AY072727) of the gene. GenBank accession numbers are provided for each sequence used. The bootstrap consensus tree was inferred from 1,000 replicates. Branches corresponding to partitions that were reproduced in less than 50% of bootstrap replicates are collapsed. The bootstrap values are indicated at the branch points. The evolutionary distances were computed using the Kimura 2-parameter method. The rate variation among sites was modelled with a gamma distribution (shape parameter = 2). No outgroup taxa was used as beta-giardin is a *Giardia*-specific structural protein.

Fig 2 shows the evolutionary relationship of the *G*. *duodenalis* isolates obtained in this study and appropriate reference sequences obtained from GenBank at the *BG* locus inferred by neighbour-joining analysis. As expected, assemblage B isolates clustered together in a well-defined, highly conserved clade, whereas sub-assemblages AII and AIII also formed distinct groups on the phylogenetic tree.

### Patients with refractory giardiasis

Molecular genotyping data were available for two patients presenting refractory giardiasis. One of them, a 55 years-old Spanish female, was initially treated with 500 mg metrodidazole twice a day for seven days, changing to a combined therapy based on 400 mg albendazole twice a day for seven days plus metronidazole at the same dosage and administration regimen described above. Prescribed treatments were proved unsuccessful in eradicating the infection, as demonstrated by the detection of the parasite in three consecutive stool samples collected during a three-month period. All three isolates were assigned to the sub-assemblages AII/AIII of *G*. *duodenalis*. The second patient was a 48 years-old Chilean man with a severe immunosuppression by AIDS who was treated with 250 mg metronidazole three times a day for seven days. Two consecutive samples collected in a two-week period were *Giardia*-positive. In both cases the infection was caused by the assemblage B of the parasite. Unfortunately this patient died soon after as a result of his poor health condition.

## Discussion

*G*. *duodenalis* is the most commonly reported enteric protozoa worldwide [3]. A total of 16,368 confirmed cases of giardiasis were documented In the European Union (EU) in 2012 [26]. Contrary to most EU countries, giardiasis is not a compulsory notifiable disease in Spain, where 859 confirmed cased were voluntary reported in that year by a network of clinical laboratories covering a quarter of the total population [9,26]. Taken together, these data strongly suggest that the actual burden of giardiasis in Spain must be far higher than available official figures show. Supporting this notion, we have conducted an epidemiological and molecular investigation on 184 cases presumptively diagnosed with giardiasis in two major public hospitals in Madrid (Central Spain) during a 14-month period. Our diagnostic analysis allowed the confirmation of the infection in 178 patients. In line with the trends found in other countries [26,27], the highest case rate of symptomatic giardiasis was observed in the age group 0–4 years, confirming that toddlers and young children are the most susceptible population to the infection. Giardiasis is frequently reported in immigrants and returning travellers from endemic regions [15–18,28], indicating that these social groups may account for a significant number of the infections detected in hospital settings. This does not seem to be the case in our study, as the vast majority (92.7%) of the cases were nationals with no relevant record of traveling abroad, whereas foreigner cases were well-stablished residents in the Madrid area. This information demonstrates that the clinical population under study corresponded to autochthonous cases of human giardiasis.

Regarding clinical data, diarrhoea, abdominal pain, and nausea/vomit were the manifestations more commonly reported, as widely documented in case record of patients with symptomatic giardiasis [29]. The occasional opportunistic nature of *G*. *duodenalis* was suggested by the finding that a proportion of the cases corresponded to immunocompromised patients including those with immunological disorders, cancer, diabetes, and HIV infections. By no means this necessarily implies that all individuals with immunodeficiency conditions were more likely to have giardiasis. For instance, whereas only 1.7% of the *G*. *duodenalis* infections were detected in diabetic patients, diabetes affects 8.7% of the total Spanish population. Similarly, a number of concurrent infections of *G*. *duodenalis* with other bacterial and parasitic pathogens were also identified. Although not fully understood, there is increasing epidemiological evidence suggesting that concomitant infections with multiple enteric pathogens may influence the extent by which *G*. *duodenalis* is able to modulate the host immune system, and, therefore, the virulence and ultimate outcome of the infection. Thus, *G*. *duodenalis* infection has been associated to different degrees (including case-control studies) to infections with *Cryptosporidium* spp. [30], *Blastocystis* spp. [31], *Salmonella* [32], *Campylobacter jejuni* [33], and *Helicobacter pylori* [34–37], all of them identified in some of the cases analysed in our study. More research should be conducted in this area in order to elucidate the physiological and immunological mechanisms responsible for this phenomenon.

Detection of *G*. *duodenalis* in most clinical laboratories still relies on concentration and CM methods [38], although rapid diagnostic assays including ICT are increasingly used in hospital settings because of their simplicity and short test time. Notably, the diagnostic performance of both CM and ICT is compromised by low sensitivities when stool samples with low parasite numbers are tested. ICT provides comparable sensitivities and specificities to CM and ELISA [39,40], but only detects half of PCR-positive samples at best [41]. In addition, a number of evaluation studies have revealed variable rates of ICT false-negative and false-positive results [42,43]. In our set of samples we estimated an ICT false-positive rate of nearly 5%, clearly indicating that ICT must be interpreted with caution and may require confirmation by a more sensitive and specific technique in certain cases. In this regard our results also demonstrated that, based on its superior sensitivity compared to DFAT, real-time PCR should be considered as the method of choice for the detection of *G*. *duodenalis* in faecal material.

Possible correlation between *G*. *duodenalis*-related clinical manifestations and genetic variants of the parasite has been investigated in a number of molecular epidemiological studies targeting human populations belonging to different age groups and geographical regions including Spain. In most of these surveys assemblage B has been mainly associated to asymptomatic infections in apparently healthy subjects [10,11,44–48]. However, opposite results identifying assemblage B as the most prevalent *G*. *duodenalis* genetic variant in infected individuals with acute or persistent diarrhoea have been documented in other surveys [49,50] including a recent case-control study [51]. Assemblage B was also the *G*. *duodenalis* assemblage more frequently found in subjects with clinical giardiasis in the present survey, accounting for 74.2% of all the isolates typed. Interestingly, this very same genetic variant was the most prevalent assemblage found both in asymptomatic children attending day care centres [11] and sheltered dogs [52] in the Madrid area. Considering these data we therefore envisage an epidemiological scenario in which B isolates are outnumbering A isolates in a 3:1 (or even larger) proportion explaining, at least partially, the predominance of assemblage B in both symptomatic and asymptomatic cases. However, this hypothesis must be confirmed in further studies with larger panels of subjects, particularly in the case of individuals with sub-clinical infections. It would be also interesting to demonstrate the potential role of infected dogs as natural reservoir of human giardiasis in this Spanish geographical region.

Our MLG analyses also revealed interesting data. Successful amplification rates for the *GDH* and *BG* loci were of 60.6% and 47.3%, respectively, well in the expected range given the single-copy nature of these genes compared to the multi-copy *SSU* rRNA gen used in the real-time PCR detection assay. Higher amplification rates at the *GDH* locus compared to the *BG* locus might be explained, at least partially, by the primer set designs and the genetic structure of target fragments. In this regard, mismatches in the binding regions of primer sequences might result in PCR amplification failure for some *G*. *duodenalis* isolates. BIV and AII were the *G*. *duodenalis* sub-types more represented. These results, together with the absence of the animal-specific assemblages (e.g. C-F), suggest that transmission of giardiasis in the clinical population under study appears to be predominantly anthroponotic in origin. An exception to this rule of thumb seems to be the finding of a single isolate assigned to sub-assemblage AI, known to be predominantly circulating in livestock and companion animal species [8] and, therefore, compatible with a zoonotic transmission event. Consistent with an epidemiological scenario of low infection pressure in a hypoendemic area, discordant genotype results AII/AIII or BIII/BIV were identified only in 10.5% of the total sub-typed isolates. Interestingly, the characterization of 11 *G*. *duodenalis* isolates as AII or AIII at the *GDH* and *BG* markers, respectively, is suggestive of an exchange of genetic material between the homologous DNA sequences of both sub-assemblages. This possibility is strengthened by the fact that only clear, unambiguous sequencing data were produced at both loci. Additionally, *G*. *duodenalis* sub-assemblages AII and AIII differ from each other by only two nucleotides at the sequence stretches considered in this study. Taken together, these data highlight the relevance of adopting MLG approaches to detect the occurrence of allelic heterogeneity and genetic recombination at the sub-assemblage level in large population surveys.

In line with previous molecular studies [53,54], we found extremely marked differences in polymorphism rates between *G*. *duodenalis* assemblage A and B isolates both at the *GDH* and *BG* genes. Whereas highly conserved nucleotide sequences with very few SNPs were observed in sub-assemblage AII and AIII sequences, a large number of well-defined point-mutations and double peaks (generally indicative of heterozygous sites) were identified in B isolates. Particularly striking was the finding that virtually all B isolate analysed at the *BG* locus differed by one to thirteen SNPs when compared with the appropriate reference sequence. Additionally, the total numbers of SNPs and SNPs inducing amino acid changes at the protein level were ~1.6 and 4.7 times higher than for sub-assemblages BIII and BIV at the *GDH* locus, respectively. There is then little surprise that some authors, based on the elevated degree of genomic diversity, protein coding capacity, and evolutionary history observed, have proposed that assemblages A and B should indeed be regarded as distinct, independent *Giardia* species [55–57].

From a biological and molecular point of view two plausible mechanisms have been suggested to explain the elevated genetic variability at the nucleotide level observed in *G*. *duodenalis* assemblage B, including true mixed infections and allelic sequence heterozygosity (ASH). Concurrent infections with different *G*. *duodenalis* assemblages or sub-assemblages may occur as a consequence of environmental mixing (e.g. ingestion of contaminated water) or re-infection with a new *G*. *duodenalis* variant [54]. Conventionally, *Giardia* has been assumed to be an organism strictly asexual [1]. Under this model, the two allelic gene copies at a given locus are expected to become highly divergent over time as a result of the independent accumulation of mutations in the absence of Mendelian segregation. However, the asexual nature of *Giardia* has been recently challenged by genomic evidence demonstrating the existence of genetic exchange by means of intragenic recombination [58], exchange of alleles [59] or even nuclear fusion within cysts [60]. Furthermore, recombination events have been described both at cellular [61] and population [62,63] levels, providing epidemiological and experimental support in favour of the existence of sexual reproduction in *Giardia*. Indeed, the high level of sequence heterogeneity (particularly in the form of double peaks) in *G*. *duodenalis* assemblage B described here and in many other molecular epidemiological studies has been interpreted as the direct consequence of a recent (although rare) sexual event in the evolutionary history of this particular lineage [64]. In this regard, it has also been suggested that genomes of the parasite undergoing recombination may become more or less virulent than the parental cells, an attractive possibility with important epidemiological implications [64].

A potential limitation of this study was the interpretation of the discordant genotype results AII/AIII and BIII/BIV obtained in our MLG analyses at the *GDH* and *BG* loci. Based on our sequencing data we favoured the hypothesis that recombination is the most likely explanation for this phenomenon. Indeed, intra-assemblage recombination has been demonstrated in AII and B isolates in a highly endemic area in Peru in which infection with multiple *G*. *duodenalis* isolates at the same time were common [62,63]. In this regard, adoption of new strategies (e.g. incorporation of additional PCR-based typing markers to our MLG scheme, sequence analysis of multiple cloned PCR products, or bioinformatics tools to assess recombination rates from population genetic data) would be highly desirable to enable unambiguous differentiation between true mixed infections and ASH events within *G*. *duodenalis* sub-assemblages.

There has been a steady increase in the number of refractory giardiasis cases reported in hospital settings in recent years [20,21,65–67]. For instance nitroimidazole-based treatment failed in 15.1% of returned travellers with giardiasis attended in a major public hospital in London (UK) in 2008, increasing to 20.6% in 2011 and to 40.2% in 2013 [65]. Overall, 69.9% of these patients were returning travellers from India [65]. Interestingly, three members of a Spanish family returning from a holiday trip to India had tinidazole-refractory giardiasis caused by the assemblage B of the parasite [21]. In our study we have identified two patients presenting refractory giardiasis, one harbouring a mixed infection AII+AIII and the other infected with assemblage B. More research should be conducted to elucidate the potential correlation between *G*. *duodenalis* assemblages/sub-assemblages and failure to conventional chemotherapeutical treatments.

## Conclusion

Human giardiasis represents an ongoing public health concern in the Madrid area and, very likely, in other Spanish regions. In order to uncover the real burden of the disease and minimize its incidence measures including enacting supportive regulations, improving the national surveillance system, and allocating more resources to research, diagnosis, and treatment must be implemented. This is the largest multi-locus genotyping survey of *G*. *duodenalis* isolates from patients with clinical manifestations conducted in Spain to date. Obtained typing and sub-typing results are consistent with those previously reported in similar studies demonstrating a large genetic variability in *G*. *duodenalis* assemble B, but no assemblage A, isolates. Our results also provide molecular epidemiological evidence at the population level in support of the existence of sexual reproduction within assemblages of *G*. *duodenalis*.

## Supporting Information

S1 Table Correlation of obtained results by conventional microscopy (CM) and immunochromatographic test (ICT) in stool samples from patients with clinical presentation compatible with giardiasis. (DOCX)

S2 Table Correlation of obtained results by direct fluorescent antibody test (DFAT) and real-time PCR in stool samples from patients with suspected^1^ *G*. *duodenalis* infections. (DOCX)

S3 Table Multilocus genotyping results of *G*. *duodenalis* isolates (*n* = 124) at the glutamate dehydrogenase (*GDH*) and beta giardin (*BG*) genes. Cycle threshold (Ct) values previously obtained by real-time PCR are indicated.
(DOCX)

S4 Table Diversity and frequency of single-nucleotide polymorphisms displayed by conflicting genotype results of sub-assemblages BIII/BIV of *Giardia duodenalis* at the glutamate dehydrogenase locus (partial sequence between positions 40 to 446) identified in the present study . Sequence AF069059 (BIII) has been used as reference.
(DOCX)

S5 Table Diversity and frequency of single-nucleotide polymorphisms displayed by conflicting genotype results of sub-assemblages BIII/BIV of *Giardia duodenalis* at the glutamate dehydrogenase locus (partial sequence between positions 76 to 482) identified in the present study . Sequence L40508 (BIV) has been used as reference.
(DOCX)

## References

[1] Adam RD. Biology of *Giardia lamblia* . Clin Microbiol Rev. 2001;14: 447–475. 11432808 10.1128/CMR.14.3.447-475.2001 PMC88984

[2] Berkman DS, Lescano AG, Gilman RH, Lopez SL, Black MM. Effects of stunting, diarrhoeal disease, and parasitic infection during infancy on cognition in late childhood: a follow-up study. Lancet 2002;359: 564–571. 11867110 10.1016/S0140-6736(02)07744-9

[3] Fletcher SM, Stark D, Harkness J, Ellis J. Enteric protozoa in the developed world: a public health perspective. Clin Microbiol Rev. 2012;25: 420–449. 10.1128/CMR.05038-11 22763633 PMC3416492

[4] Baldursson S, Karanis P. Waterborne transmission of protozoan parasites: review of worldwide outbreaks—an update 2004–2010. Water Res. 2011;45: 6603–6614. 10.1016/j.watres.2011.10.013 22048017

[5] Robertson LJ. Documented foodborne outbreaks of giardiasis In: Hartel RW, editor. Giardia as a foodborne pathogen. New York: SpringerBriefs; 2013 pp. 13–17.

[6] Ballweber LR, Xiao L, Bowman DD, Kahn G, Cama VA. Giardiasis in dogs and cats: update on epidemiology and public health significance. Trends Parasitol. 2010;26: 180–189. 10.1016/j.pt.2010.02.005 20202906

[7] Feng Y, Xiao L. Zoonotic potential and molecular epidemiology of *Giardia* species and giardiasis. Clin Microbiol Rev. 2011;24: 110–140. 10.1128/CMR.00033-10 21233509 PMC3021202

[8] Ryan U, Cacciò SM. Zoonotic potential of *Giardia* . Int J Parasitol. 2013;43: 943–956. 10.1016/j.ijpara.2013.06.001 23856595

[9] Carmena D, Cardona GA, Sánchez-Serrano LP. Current situation of *Giardia* infection in Spain: Implications for public health. World J Clin Infect Dis. 2012;2: 1–12.

[10] Cardona GA, Carabin H, Goñi P, Arriola L, Robinson G, Fernández-Crespo JC, et al Identification and molecular characterization of *Cryptosporidium* and *Giardia* in children and cattle populations from the province of Álava, North of Spain. Sci Total Environ. 2011;412–413: 101–108. 10.1016/j.scitotenv.2011.09.076 22030246

[11] Mateo M, Mateo M, Montoya A, Bailo B, Saugar JM, Aguilera M, et al Detection and molecular characterization of Giardia duodenalis in children attending day care centers in Majadahonda, Madrid, Central Spain. Medicine (Baltimore) 2014;93: e75. 25275524 10.1097/MD.0000000000000075 PMC4616291

[12] Rodríguez-Hernández J, Canut-Blasco A, Martín-Sánchez AM. Seasonal prevalences of Cryptosporidium and Giardia infections in children attending day care centres in Salamanca (Spain) studied for a period of 15 months. Eur J Epidemiol. 1996;12: 291–295. 8884197 10.1007/BF00145419

[13] González-Moreno O, Domingo L, Teixidor J, Gracenea M. Prevalence and associated factors of intestinal parasitisation: a cross-sectional study among outpatients with gastrointestinal symptoms in Catalonia, Spain. Parasitol Res. 2011;108: 87–93. 10.1007/s00436-010-2044-2 20862495

[14] Goñi P, Aldana DE, Clavel A, Seral C, Remacha MA, Castillo FJ. Prevalence of Giardia duodenalis assemblage B in humans in Zaragoza and León, Spain. Enferm Infecc Microbiol Clin. 2010;28: 710–712. 20934781 10.1016/j.eimc.2010.04.010

[15] Cobo F, Salas-Coronas J, Cabezas-Fernández MT, Vázquez-Villegas J, Cabeza-Barrera MI, Soriano-Pérez MJ. Infectious diseases in immigrant population related to the time of residence in Spain. J Immigr Minor Health. In press. 10.1007/s10903-014-0141-5 25466580

[16] López-Vélez R, Huerga H, Turrientes MC. Infectious diseases in immigrants from the perspective of a tropical medicine referral unit. Am J Trop Med Hyg 2003;69: 115–121. 12932108

[17] Manzardo C, Treviño B, Gómez i Prat J, Cabezos J, Monguí E, Clavería I, et al Communicable diseases in the immigrant population attended to in a tropical medicine unit: epidemiological aspects and public health issues. Travel Med Infect Dis. 2008;6: 4–11. 10.1016/j.tmaid.2007.11.002 18342267

[18] Soriano JM, Domènech G, Martínez MC, Mañes J, Soriano F. Intestinal parasitic infections in hosted Saharawi children. Trop Biomed. 2011;28: 557–562. 22433884

[19] Alonso-Sanz M, Chaves F, Dronda F, Catalán S, González-López A. Intestinal parasitoses in the prison population in the Madrid area (1991–1993). Enferm Infecc Microbiol Clin. 1995;13: 90–95. 7794345

[20] Muñoz J, Aldasoro E, Requena A, Comin AM, Pinazo MJ, Bardají A, et al Refractory giardiasis in Spanish travellers. Travel Med Infect Dis. 2013;11: 126–129. 10.1016/j.tmaid.2012.10.004 23218784

[21] Requena-Méndez A, Goñi P, Lóbez S, Oliveira I, Aldasoro E, Valls ME, et al A family cluster of giardiasis with variable treatment responses: refractory giardiasis in a family after a trip to India. Clin Microbiol Infect. 2014;20: O135–O138. 10.1111/1469-0691.12327 23926944

[22] Verweij JJ, Schinkel J, Laeijendecker D, van Rooyen MA, van Lieshout L, Polderman AM. Real-time PCR for the detection of *Giardia lamblia* . Mol Cell Probes 2003;17: 223–225. 14580396 10.1016/s0890-8508(03)00057-4

[23] Read CM, Monis PT, Thompson RC. Discrimination of all genotypes of *Giardia duodenalis* at the glutamate dehydrogenase locus using PCR-RFLP. Infect Genet Evol. 2004;4: 125–130. 15157630 10.1016/j.meegid.2004.02.001

[24] Lalle M, Pozio E, Capelli G, Bruschi F, Crotti D, Cacciò SM. Genetic heterogeneity at the beta-giardin locus among human and animal isolates of *Giardia duodenalis* and identification of potentially zoonotic subgenotypes. Int J Parasitol. 2005;35: 207–213. 15710441 10.1016/j.ijpara.2004.10.022

[25] Tamura K, Stecher G, Peterson D, Filipski A, Kumar S. MEGA6: Molecular Evolutionary Genetics Analysis version 6.0. Mol Biol Evol. 2013;30: 2725–2729. 10.1093/molbev/mst197 24132122 PMC3840312

[26] European Centre for Disease Prevention and Control. Annual epidemiological report 2014 –food- and waterborne diseases and zoonoses. Stockholm: ECDC; 2014.

[27] Barry MA, Weatherhead JE, Hotez PJ, Woc-Colburn L. Childhood parasitic infections endemic to the United States. Pediatr Clin North Am. 2013;60: 471–485. 10.1016/j.pcl.2012.12.011 23481112

[28] Gautret P, Cramer JP, Field V, Caumes E, Jensenius M, Gkrania-Klotsas E, et al Infectious diseases among travellers and migrants in Europe, EuroTravNet 2010. Euro Surveill. 2012;17. 22790534

[29] Farthing MJ. Giardiasis. Gastroenterol Clin North Am. 1996;25: 493–515. 8863037 10.1016/s0889-8553(05)70260-0

[30] Wang L, Xiao L, Duan L, Ye J, Guo Y, Guo M, et al Concurrent infections of Giardia duodenalis, Enterocytozoon bieneusi, and Clostridium difficile in children during a cryptosporidiosis outbreak in a pediatric hospital in China. PLoS Negl Trop Dis. 2013;7: e2437 10.1371/journal.pntd.0002437 24069491 PMC3772047

[31] Elghareeb AS, Younis MS, El Fakahany AF, Nagaty IM, Nagib MM. Laboratory diagnosis of Blastocystis spp. in diarrheic patients. Trop Parasitol. 2015;5: 36–41. 10.4103/2229-5070.149919 25709951 PMC4326992

[32] Oberhelman RA, Flores-Abuxapqui J, Suarez-Hoil G, Puc-Franco M, Heredia-Navarrete M, Vivas-Rosel M, et al Asymptomatic salmonellosis among children in day-care centers in Mérida, Yucatan, Mexico. Pediatr Infect Dis J. 2001;20: 792–797. 11734743 10.1097/00006454-200108000-00014

[33] Krumkamp R, Sarpong N, Schwarz NG, Adlkofer J, Loag W, Eibach D, et al Gastrointestinal infections and diarrheal disease in Ghanaian infants and children: an outpatient case-control study. PLoS Negl Trop Dis. 2015;9: e0003568 10.1371/journal.pntd.0003568 25738935 PMC4349824

[34] Zeyrek D, Zeyrek F, Cakmak A, Cekin A. Association of *Helicobacter pylori* and giardiasis in children with recurrent abdominal pain. Turkiye Parazitol Derg. 2008;32: 4–7. 18351542

[35] Ankarklev J, Hestvik E, Lebbad M, Lindh J, Kaddu-Mulindwa DH, Andersson JO, et al Common coinfections of *Giardia intestinalis* and *Helicobacter pylori* in non-symptomatic Ugandan children. PLoS Negl Trop Dis. 2012;6: e1780 10.1371/journal.pntd.0001780 22953010 PMC3429385

[36] Júlio C, Vilares A, Oleastro M, Ferreira I, Gomes S, Monteiro L, et al Prevalence and risk factors for Giardia duodenalis infection among children: a case study in Portugal. Parasit Vectors. 2012;5: 22 10.1186/1756-3305-5-22 22284337 PMC3275531

[37] Eldash HH, Bekhit OE, Algameel AA. Impact of *Helicobacter pylori*-giardiasis coinfection on children with recurrent abdominal pain. J Egypt Soc Parasitol. 2013;43: 509–516. 24260829 10.12816/0006407

[38] Manser M, Granlund M, Edwards H, Saez A, Petersen E, Evengard B, et al Detection of *Cryptosporidium* and *Giardia* in clinical laboratories in Europe—a comparative study. Clin Microbiol Infect. 2014;20: O65–O71. 10.1111/1469-0691.12297 24033667

[39] Gutiérrez-Cisneros MJ, Martínez-Ruiz R, Subirats M, Merino FJ, Millán R, Fuentes I. Assessment of two commercially available immunochromatographic assays for a rapid diagnosis of Giardia duodenalis and Cryptosporidium spp. in human fecal specimens. Enferm Infecc Microbiol Clin. 2011;29: 201–203. 21342732 10.1016/j.eimc.2010.09.005

[40] Goñi P, Martín B, Villacampa M, García A, Seral C, Castillo FJ, et al Evaluation of an immunochromatographic dip strip test for simultaneous detection of *Cryptosporidium* spp, *Giardia duodenalis*, and *Entamoeba histolytica* antigens in human faecal samples. Eur J Clin Microbiol Infect Dis. 2012;31: 2077–2082. 10.1007/s10096-012-1544-7 22262367

[41] Ignatius R, Gahutu JB, Klotz C, Musemakweri A, Aebischer T, Mockenhaupt FP. Detection of *Giardia duodenalis* assemblage A and B isolates by immunochromatography in stool samples from Rwandan children. Clin Microbiol Infect. 2014;20: O783–O785. 10.1111/1469-0691.12596 24533695

[42] Garcia LS, Shimizu RY, Novak S, Carroll M, Chan F. Commercial assay for detection of *Giardia lamblia* and *Cryptosporidium parvum* antigens in human fecal specimens by rapid solid-phase qualitative immunochromatography. J Clin Microbiol. 2003;41: 209–212. 12517850 10.1128/JCM.41.1.209-212.2003 PMC149610

[43] Nguyen TK, Kherouf H, Blanc-Pattin V, Allais E, Chevalier Y, Richez A, et al Evaluation of an immunochromatographic assay: Giardia-Strip^®^ (Coris BioConcept) for detection of *Giardia intestinalis* in human fecal specimens. Eur J Clin Microbiol Infect Dis. 2012;31: 623–625. 10.1007/s10096-011-1332-9 21732200

[44] Read C, Walters J, Robertson ID, Thompson RC. Correlation between genotype of *Giardia duodenalis* and diarrhoea. Int J Parasitol. 2002;32: 229–231. 11812501 10.1016/s0020-7519(01)00340-x

[45] Haque R, Mondal D, Karim A, Molla IH, Rahim A, Faruque AS, et al Prospective case—control study of the association between common enteric protozoal parasites and diarrhea in Bangladesh. Clin Infect Dis. 2009;48: 1191–1197. 10.1086/597580 19323634 PMC2883291

[46] Almeida AA, Delgado ML, Soares SC, Castro AO, Moreira MJ, Mendonça CM, et al Genotype analysis of *Giardia* isolated from asymptomatic children in northern Portugal. J Eukaryot Microbiol. 2006;53: S177–S178. 17169051 10.1111/j.1550-7408.2006.00222.x

[47] Sahagún J, Clavel A, Goñi P, Seral C, Llorente MT, Castillo FJ, et al Correlation between the presence of symptoms and the *Giardia duodenalis* genotype. Eur J Clin Microbiol Infect Dis. 2008;27: 81–83. 17943329 10.1007/s10096-007-0404-3

[48] Aydin AF, Besirbellioglu BA, Avci IY, Tanyuksel M, Araz E, Pahsa A. Classification of *Giardia duodenalis* parasites in Turkey into groups A and B using restriction fragment length polymorphism. Diagn Microbiol Infect Dis. 2004;50: 147–151. 15474326 10.1016/j.diagmicrobio.2004.06.001

[49] Homan WL, Mank TG. Human giardiasis: genotype linked differences in clinical symptomatology. Int J Parasitol. 2001;31: 822–826. 11403774 10.1016/s0020-7519(01)00183-7

[50] Flecha MJ, Benavides CM, Tissiano G, Tesfamariam A, Cuadros J, de Lucio A, et al Detection and molecular characterisation of *Giardia duodenalis*, *Cryptosporidium* spp. and *Entamoeba* spp. among patients with gastrointestinal symptoms in Gambo Hospital, Oromia Region, southern Ethiopia. Trop Med Int Health. 2015;20: 1213–1222. 10.1111/tmi.12535 25939247

[51] Minetti C, Lamden K, Durband C, Cheesbrough J, Platt K, Charlett A, et al Case-control study of risk factors for sporadic giardiasis and parasite assemblages in North West England. J Clin Microbiol. In press. 10.1128/JCM.00715-15 PMC4572545 26157151

[52] Dado D, Montoya A, Blanco MA, Miró G, Saugar JM, Bailo B, et al Prevalence and genotypes of *Giardia duodenalis* from dogs in Spain: possible zoonotic transmission and public health importance. Parasitol Res. 2012; 111:2419–2422. 10.1007/s00436-012-3100-x 22983168

[53] Lebbad M, Ankarklev J, Tellez A, Leiva B, Andersson JO, Svärd S. Dominance of Giardia assemblage B in León, Nicaragua. Acta Trop. 2008;106: 44–53. 10.1016/j.actatropica.2008.01.004 18325480

[54] Sprong H, Cacciò SM, van der Giessen JW. Identification of zoonotic genotypes of Giardia duodenalis. PLoS Negl Trop Dis. 2009;3: e558 10.1371/journal.pntd.0000558 19956662 PMC2777335

[55] Adam RD, Dahlstrom EW, Martens CA, Bruno DP, Barbian KD, Ricklefs SM, et al Genome sequencing of Giardia lamblia genotypes A2 and B isolates (DH and GS) and comparative analysis with the genomes of genotypes A1 and E (WB and Pig). Genome Biol Evol. 2013;5: 2498–2511. 10.1093/gbe/evt197 24307482 PMC3879983

[56] Jerlström-Hultqvist J, Ankarklev J, Svärd SG. Is human giardiasis caused by two different *Giardia* species? Gut Microbes. 2010;1: 379–382. 10.4161/gmic.1.6.13608 21468219 PMC3056102

[57] Nash TE, McCutchan T, Keister D, Dame JB, Conrad JD, Gillin FD. Restriction-endonuclease analysis of DNA from 15 *Giardia* isolates obtained from humans and animals. J Infect Dis. 1985 152: 64–73. 2409186 10.1093/infdis/152.1.64

[58] Kosuwin R, Putaporntip C, Pattanawong U, Jongwutiwes S. Clonal diversity in *Giardia duodenalis* isolates from Thailand: evidences for intragenic recombination and purifying selection at the beta giardin locus. Gene. 2010;449: 1–8. 10.1016/j.gene.2009.09.010 19796671

[59] Teodorovic S, Braverman JM, Elmendorf HG. Unusually low levels of genetic variation among *Giardia lamblia* isolates. Eukaryot Cell. 2007;6: 1421–30. 17557879 10.1128/EC.00138-07 PMC1951139

[60] Poxleitner MK, Carpenter ML, Mancuso JJ, Wang CJ, Dawson SC, Cande WZ. Evidence for karyogamy and exchange of genetic material in the binucleate intestinal parasite *Giardia intestinalis* . Science. 2008;319: 1530–1533. 10.1126/science.1153752 18339940

[61] Ankarklev J, Svärd SG, Lebbad M. Allelic sequence heterozygosity in single *Giardia* parasites. BMC Microbiol. 2012;12: 65 10.1186/1471-2180-12-65 22554281 PMC3438080

[62] Cooper MA, Adam RD, Worobey M, Sterling CR. Population genetics provides evidence for recombination in *Giardia* . Curr Biol. 2007;17: 1984–1988. 17980591 10.1016/j.cub.2007.10.020

[63] Cooper MA, Sterling CR, Gilman RH, Cama V, Ortega Y, Adam RD. Molecular analysis of household transmission of *Giardia lamblia* in a region of high endemicity in Peru. J Infect Dis. 2010;202: 1713–1721. 10.1086/657142 20977340 PMC2974043

[64] Andersson JO. Double peaks reveal rare diplomonad sex. Trends Parasitol. 2012;28: 46–52. 10.1016/j.pt.2011.11.002 22192817

[65] Nabarro LE, Lever RA, Armstrong M, Chiodini PL. Increased incidence of nitroimidazole-refractory giardiasis at the Hospital for Tropical Diseases, London: 2008–2013. Clin Microbiol Infect. 2015;21: 791–796. 10.1016/j.cmi.2015.04.019 25975511

[66] Mørch K, Hanevik K, Robertson LJ, Strand EA, Langeland N. Treatment-ladder and genetic characterisation of parasites in refractory giardiasis after an outbreak in Norway. J Infect. 2008;56:268–273. 10.1016/j.jinf.2008.01.013 18328567

[67] Yadav P, Tak V, Mirdha BR, Makharia GK. Refractory giardiasis: a molecular appraisal from a tertiary care centre in India. Indian J Med Microbiol. 2014;32: 378–382. 10.4103/0255-0857.142236 25297020

