# Supplementary material for: Molecular Genotyping of Giardia duodenalis Isolates from Symptomatic Individuals Attending Two Major Public Hospitals in Madrid, Spain
Source: PLoS One. 2015 Dec 7;10(12):e0143981. doi: 10.1371/journal.pone.0143981 (PMC4671680; doi:10.1371/journal.pone.0143981)
Supplement: S1 Table — (DOCX) [file pone.0143981.s001.docx]

**S1 Table**

| **Test combinations** | **Number of samples** | **Percentage** |
| --- | --- | --- |
| CM (+) and ICT (+) | 132 | 86.8% |
| CM (+) and ICT (–) | 7 | 4.6% |
| CM (–) and ICT (+) | 13 | 8.6% |
| CM (–) and ICT (–) | 0 | 0.0 |
| Total | 152 | 100 |
