# Supplementary material for: Molecular Genotyping of Giardia duodenalis Isolates from Symptomatic Individuals Attending Two Major Public Hospitals in Madrid, Spain
Source: PLoS One. 2015 Dec 7;10(12):e0143981. doi: 10.1371/journal.pone.0143981 (PMC4671680; doi:10.1371/journal.pone.0143981)
Supplement: S2 Table — (DOCX) [file pone.0143981.s002.docx]

**S2 Table**

| **Test combinations** | **Number of samples** | **Percentage** |
| --- | --- | --- |
| DFAT (+) and real-time PCR (+) | 147 | 73.9 |
| DFAT (+) and real-time PCR (–) | 1 | 0.5 |
| DFAT (–) and real-time PCR (+) | 33 | 16.6 |
| DFAT (–) and real-time PCR (–) | 10 | 5.0 |
| Real-time PCR (+) only | 8 | 4.0 |
| Total | 199 | 100 |

^1^As determined by conventional microscopy and/or immunochromatographic test.
