# Supplementary material for: Molecular Genotyping of Giardia duodenalis Isolates from Symptomatic Individuals Attending Two Major Public Hospitals in Madrid, Spain
Source: PLoS One. 2015 Dec 7;10(12):e0143981. doi: 10.1371/journal.pone.0143981 (PMC4671680; doi:10.1371/journal.pone.0143981)
Supplement: S3 Table — Cycle threshold (Ct) values previously obtained by real-time PCR are indicated. (DOCX) [file pone.0143981.s003.docx]

**S3 Table**

| **Isolate** | **Hospital** | **Ct value** | **Genotyping results** | | |
| --- | --- | --- | --- | --- | --- |
|  |  |  | ***GDH* locus** | ***BG* locus** | **Assigned genotype** |
| 285_13 | UHSO | 23.0 | BIII | B | BIII |
| 297_13 | UHPHM | 28.2 | BIV | – | BIV |
| 2_14 | UHSO | 21.9 | BIV | B | BIV |
| 17_14 | UHSO | 25.7 | BIV | B | BIV |
| 26_14 | UHPHM | 23.6 | BIV | – | BIV |
| 29_14 | UHPHM | 27.9 | BIV | – | BIV |
| 32_14 | UHSO | 25.4 | AII | AII | AII |
| 38_14 | UHPHM | 28.8 | AI | – | AI |
| 39_14 | UHPHM | 27.9 | AII | AII | AII |
| 52_14 | UHPHM | 20.0 | BIV | – | BIV |
| 55_14 | UHPHM | 25.7 | AII | – | AII |
| 63_14 | UHPHM | 25.0 | AII | – | AII |
| 64_14 | UHPHM | 27.0 | BIV | – | BIV |
| 66_14 | UHPHM | 26.4 | BIV | – | BIV |
| 67_14 | UHPHM | 22.0 | AII | – | AII |
| 143_14 | UHPHM | 23.9 | BIV | B | BIV |
| 144_14 | UHPHM | 27.6 | BIV | – | BIV |
| 145_14 | UHPHM | 29.5 | BIV | B | BIV |
| 215_14 | UHSO | 21.0 | – | B | B |
| 224_14 | UHPHM | 24.9 | – | B | B |
| 226_14 | UHPHM | 24.1 | BIV | B | BIV |
| 233_14 | UHPHM | 24.8 | BIV | B | BIV |
| 235_14 | UHPHM | 28.3 | BIV | – | BIV |
| 236_14 | UHSO | 23.2 | BIV | B | BIV |
| 239_14 | UHPHM | 25.5 | BIV | B | BIV |
| 242_14 | UHPHM | 28.3 | BIV | – | BIV |
| 248_14 | UHPHM | 26.2 | BIV | – | BIV |
| 259_14 | UHSO | 25.0 | BIV | – | BIV |
| 273_14 | UHPHM | 23.0 | BIV | – | BIV |
| 274_14 | UHPHM | 25.0 | BIV | B | BIV |
| 281_14 | UHPHM | 25.3 | BIV | – | BIV |
| 284_14 | UHPHM | 21.3 | BIV | B | BIV |
| 291_14 | UHPHM | 26.5 | BIV | – | BIV |
| 294_14 | UHSO | 22.7 | BIV | B | BIV |
| 301_14 | UHPHM | 25.1 | AII | AIII | AII/AIII |
| 302_14 | UHPHM | 21.8 | BIII/BIV | B | BIII/BIV |
| 303_14 | UHPHM | 21.7 | BIV | B | BIV |
| 304_14 | UHPHM | 24.8 | BIV | B | BIV |
| 305_14 | UHSO | 21.4 | BIV | B | BIV |
| 308_14 | UHSO | 25.9 | BIV | B | BIV |
| 309_14 | UHSO | 25.2 | AII | AII | AII |
| 321_14 | UHPHM | 20.7 | BIV | B | BIV |
| 322_14 | UHPHM | 24.4 | BIV | B | BIV |
| 325_14 | UHPHM | 23.4 | BIV | B | BIV |
| 344_14 | UHSO | 26.0 | AII | AIII | AII/AIII |
| 362_14 | UHPHM | 27.7 | – | B | B |
| 363_14 | UHPHM | 24.8 | AII | AIII | AII/AIII |
| 364_14 | UHSO | 32.2 | BIV | – | BIV |
| 371_14 | UHPHM | 29.0 | BIV | – | BIV |
| 372_14 | UHPHM | 26.7 | BIII | – | BIII |
| 376_14 | UHPHM | 23.9 | BIV | B | BIV |
| 377_14 | UHPHM | 25.6 | AII | – | AII |
| 379_14 | UHPHM | 25.5 | BIV | – | BIV |
| 381_14 | UHPHM | 20.0 | BIV | B | BIV |
| 382_14 | UHPHM | 30.0 | AII | AIII | AII/AIII |
| 392_14 | UHPHM | 22.0 | AII | AIII | AII/AIII |
| 393_14 | UHPHM | 23.0 | BIII | B | BIII |
| 396_14 | UHSO | 25.0 | BIV | B | BIV |
| 397_14 | UHSO | 28.0 | BIV | B | BIV |
| 413_14 | UHPHM | 24.8 | BIV | – | BIV |
| 418_14 | UHPHM | 24.8 | AII | AII | AII |
| 419_14 | UHPHM | 19.5 | BIV | B | BIV |
| 424_14 | UHPHM | 30.9 | AII | AIII | AII/AIII |
| 430_14 | UHSO | 23.5 | AII | AIII | AII/AIII |
| 436_14 | UHSO | 24.8 | BIV | B | BIV |
| 461_14 | UHSO | 25.7 | BIV | B | BIV |
| 462_14 | UHPHM | 24.4 | AII | AIII | AII/AIII |
| 463_14 | UHPHM | 25.1 | BIV | – | BIV |
| 472_14 | UHPHM | 22.1 | BIV | B | BIV |
| 473_14 | UHPHM | 23.0 | BIV | B | BIV |
| 474_14 | UHPHM | 24.2 | AII | AIII | AII/AIII |
| 475_14 | UHPHM | 23.0 | BIV | B | BIV |
| 479_14 | UHPHM | 25.0 | BIV | B | BIV |
| 486_14 | UHSO | 24.6 | BIV | B | BIV |
| 487_14 | UHSO | 21.1 | BIV | B | BIV |
| 488_14 | UHSO | 20.8 | AII | AII | AII |
| 512_14 | UHPHM | 26.9 | – | AIII | AIII |
| 513_14 | UHPHM | 24.6 | AII | AII | AII |
| 544_14 | UHSO | 20.7 | AII | AIII | AII/AIII |
| 545_14 | UHSO | 24.6 | BIV | B | BIV |
| 546_14 | UHSO | 21.1 | BIV | B | BIV |
| 549_14 | UHSO | 23.3 | – | B | BIV |
| 550_14 | UHSO | 20.9 | BIV | – | BIV |
| 561_14 | UHPHM | 23.0 | AII | AII | AII |
| 565_14 | UHPHM | 23.6 | BIV | – | BIV |
| 566_14 | UHPHM | 22.7 | BIV | – | BIV |
| 567_14 | UHPHM | 27.8 | BIV | B | BIV |
| 568_14 | UHPHM | 20.8 | BIV | B | BIV |
| 569_14 | UHPHM | 22.9 | BIII/BIV | – | BIII/BIV |
| 570_14 | UHPHM | 19.9 | BIII | – | BIII |
| 571_14 | UHPHM | 24.4 | BIV | B | BIV |
| 572_14 | UHPHM | 24.9 | AII | AII | AII |
| 574_14 | UHSO | 27.0 | BIV | – | BIV |
| 575_14 | UHSO | 23.7 | BIV | B | BIV |
| 577_14 | UHSO | 27.1 | BIV | B | BIV |
| 580_14 | UHSO | 26.1 | BIV | B | BIV |
| 581_14 | UHSO | 26.6 | AII | AII | AII |
| 582_14 | UHSO | 23.9 | BIV | B | BIV |
| 583_14 | UHSO | 30.0 | – | B | B |
| 584_14 | UHSO | 26.3 | – | B | B |
| 594_14 | UHPHM | 24.7 | – | B | B |
| 597_14 | UHSO | 21.0 | BIV | B | BIV |
| 640_14 | UHPHM | 26.0 | BIV | B | BIV |
| 641_14 | UHPHM | 23.3 | BIV | B | BIV |
| 642_14 | UHPHM | 25.9 | BIV | B | BIV |
| 643_14 | UHPHM | 22.4 | BIV | B | BIV |
| 644_14 | UHPHM | 26.7 | BIV | B | BIV |
| 658_14 | UHPHM | 26.3 | AII | AII | AII |
| 659_14 | UHPHM | 22.5 | BIV | B | BIV |
| 664_14 | UHSO | 21.2 | AII | AII | AII |
| 668_14 | UHPHM | 24.0 | BIV | B | BIV |
| 677_14 | UHPHM | 31.0 | BIV | – | BIV |
| 680_14 | UHPHM | 23.5 | AII | AII | AII |
| 681_14 | UHPHM | 26.7 | BIV | B | BIV |
| 22_15 | UHPHM | 26.9 | BIV | – | BIV |
| 23_15 | UHPHM | 26.4 | BIV | B | BIV |
| 24_15 | UHPHM | 23.3 | BIII | B | BIII |
| 25_15 | UHPHM | 21.6 | AII | AIII | AII/AIII |
| 30_15 | UHSO | 29.5 | AII | – | AII |
| 50_15 | UHPHM | 26.2 | AII | AII | AII |
| 67_15 | UHPHM | 25.6 | AII | – | AII |
| 73_15 | UHSO | 23.6 | BIV | B | BIV |
| 74_15 | UHSO | 28.9 | – | B | B |
| 75_15 | UHSO | 25.7 | – | B | B |
