# Supplementary material for: Molecular Genotyping of Giardia duodenalis Isolates from Symptomatic Individuals Attending Two Major Public Hospitals in Madrid, Spain
Source: PLoS One. 2015 Dec 7;10(12):e0143981. doi: 10.1371/journal.pone.0143981 (PMC4671680; doi:10.1371/journal.pone.0143981)
Supplement: S4 Table — . Sequence AF069059 (BIII) has been used as reference. (DOCX) [file pone.0143981.s004.docx]

**S4 Table**

|  |  | **Nucleotide at position of reference sequence AF069059 (BIII)** | | | | | | | | | | | | | |
| --- | --- | --- | --- | --- | --- | --- | --- | --- | --- | --- | --- | --- | --- | --- | --- |
|  |  | **39** | **99** | **147** | **150** | **204** | **219** | **237** | **276** | **309** | **330** | **336** | **360** | **387** | **402** |
|  |  | **C** | **C** | **T** | **G** | **C** | **T** | **T** | **T** | **C** | **C** | **C** | **C** | **C** | **G** |
| **Isolate** | **Number of isolates** |  |  |  |  |  |  |  |  |  |  |  |  |  |  |
| KT310374 | 1 | Y | Y | Y | R | . | Y | Y | Y | Y | Y | Y | Y | Y | R |
| KT310375 | 1 | T | Y | . | . | T | Y | Y | . | Y | . | Y | . | . | . |

R: A/G; Y: C/T.
