# Supplementary material for: Molecular Genotyping of Giardia duodenalis Isolates from Symptomatic Individuals Attending Two Major Public Hospitals in Madrid, Spain
Source: PLoS One. 2015 Dec 7;10(12):e0143981. doi: 10.1371/journal.pone.0143981 (PMC4671680; doi:10.1371/journal.pone.0143981)
Supplement: S5 Table — . Sequence L40508 (BIV) has been used as reference. (DOCX) [file pone.0143981.s005.docx]

**S5 Table.**

|  |  | **Nucleotide at position of reference sequence L40508 (BIV)** | | | | | | | | | | | | | | | |
| --- | --- | --- | --- | --- | --- | --- | --- | --- | --- | --- | --- | --- | --- | --- | --- | --- | --- |
|  |  | **63** | **75** | **135** | **183** | **186** | **240** | **255** | **273** | **312** | **345** | **366** | **372** | **387** | **396** | **423** | **438** |
|  |  | **C** | **C** | **T** | **T** | **G** | **C** | **C** | **C** | **T** | **C** | **T** | **C** | **T** | **C** | **C** | **A** |
| **Sub-type** | **Number of isolates** |  |  |  |  |  |  |  |  |  |  |  |  |  |  |  |  |
| KT310374 |  | T | Y | Y | Y | R | . | Y | Y | Y | Y | Y | Y | C | Y | Y | R |
| KT310375 |  | T | T | Y | . | . | T | Y | Y | . | Y | C | Y | C | . | . | G |

R: A/G; Y: C/T.
